# NADase and now Ca^2+^ channel, what else to learn about plant NLRs?

**DOI:** 10.1007/s44154-021-00007-0

**Published:** 2021-08-27

**Authors:** Li Wan, Zuhua He

**Affiliations:** National Key Laboratory of Plant Molecular Genetics, CAS Center for Excellence in Molecular Plant Sciences, Institute of Plant Physiology and Ecology, Chinese Academy of Sciences, Shanghai, China

**Keywords:** Immune receptors, NLR, Defense, Cell death, Ca^2+^ channel

## Abstract

Plant intracellular immune receptors known as NLR (Nucleotide-binding Leucine-rich repeat, NB-LRR) proteins confer resistance and cause cell death upon recognition of cognate effector proteins from pathogens. Plant NLRs contain a variable N-terminal domain: a Toll/interleukin-1 receptor (TIR) domain or a coiled-coil (CC) domain or an RPW8 (Resistance to Powdery Mildew 8)-like CC (CC^R^) domain. TIR-NLR, CC-NLR and CC^R^-NLR are known as TNL, CNL and RNL, respectively. TNLs and CNLs recognize pathogen effectors to activate cell death and defense responses, thus are regarded as sensor NLRs. RNLs are required downstream of TNLs to activate cell death and defense responses, thus are regarded as helper NLRs. Previous studies show that some TNLs form tetrameric resistosome as NAD^+^ cleaving enzymes to transduce signal, while some CNLs form pentameric resistosome with undefined biochemical function. Two recent breakthrough studies show that activated CNL and RNL function as Ca^2+^ channel to cause cell death and defense responses and provide a completely new insight into the downstream signaling events of CNL and TNL pathways.

Plants have evolved both cell surface and intracellular immune receptors to detect pathogen molecules and activate defense. Plant intracellular immune receptors known as NLRs recognize effectors secreted by pathogens into plant cells and initiate effector-triggered immunity (ETI). ETI involves strong defense gene induction and hypersensitive cell death localized at the site of infection to restrict pathogen spread. NLRs constitute an important source of resistance for breeding in agriculture (Jones et al. 2016). Investigation of the molecular mechanisms by which NLRs function to trigger immunity represents a crucial step towards the goal of engineering effective broad-spectrum resistance in crops.

TNL and CNL recognize pathogen effectors to trigger cell death and immune responses, thus are regarded as sensor NLRs (Duxbury et al. 2021). Downstream of sensor NLRs, higher plants also use helper NLRs to transduce signals. There are three described classes of helper NLRs: the NB-LRR protein required for HR-associated cell death (NRC) family, the ACTIVATED DISEASE RESISTANCE 1 (ADR1) family, and the N REQUIRED GENE 1 (NRG1) family (Jubic et al. 2019). NRCs contain the canonical CC domains at their N-termini and are a special class of CNL. In *Nicotiana benthmiana* (*Nb*), NRCs are required for phylogenetically related CNLs to activate cell death and immune responses (Wu et al. 2017). ADR1s and NRG1s, commonly present in dicots, carry an CC^R^ domain at their N-termini and belong to the RNL family (Jubic et al. 2019). In Arabidopsis, ADR1s and NRG1s function downstream of TNL to confer resistance and induce cell death, respectively (Lapin et al. 2019).

The biochemical functions of plant NLRs to activate cell death and defense remain elusive until recent breakthrough studies. The Arabidopsis CNL ZAR1 represents one of the best structurally characterized NLRs with implications in downstream signaling mechanisms (Wang et al. 2019a; Wang et al. 2019b). Pathogen effectors AvrAC and HopZ1a can induce oligomerization of ZAR1 in Arabidopsis protoplasts (Hu et al. 2020). Upon effector recognition, ZAR1 undergoes dramatic conformational changes from an inactive monomeric state to an active pentameric state with the first helix in the 4-helical-bundle(4HB) of its CC domain being flipped out, potentially forming a pore at the plasma membrane (PM) (Wang et al. 2019a). The funnel-shaped pore structure of ZAR1 resistosome is suggested to function as a channel but has not been experimentally validated until recently.

To address this question, a landmark study by Bi et al. showed that the effector activated ZAR1 resistosome protrudes into the PM and forms calcium-permeable cation channel leading to calcium influx and further activation of cell death and defense responses (Bi et al. 2021). Expression of ZAR1 along with other genetic requirements and the corresponding effector in *Xenopus oocytes* induced strong current traces upon voltage application in the two-electrode voltage-clamp assay, consistent with the suggested ZAR1 channel activity. To further investigate ZAR1 channel activity, they performed electrophysiology studies using planar lipid bilayers and reconstituted ZAR1 resistosome protein in vitro. The result confirmed that ZAR1 resistosome was inserted into planar lipid bilayers as non-selective cation channel permeable to Na^+^, K^+^, Cs^+^, Mg^2+^, and Ca^2+^. Conserved glutamic acid E11 lying in the central cavity of ZAR1 resistosome is required for ZAR1 channel activity and function. Single-molecule imaging showed that activated ZAR1 proteins form approximate pentamers accumulating in the PM. Elegant cell biology further revealed the sequential events before ZAR1-triggered cell death in Arabidopsis protoplasts such as calcium influx, accumulation of reactive oxygen species (ROS), destruction of vacuoles and chloroplasts, loss of PM integrity and eventual cell rupture (Bi et al. 2021). Hence ZAR1 functions as both sensor of pathogen and executor of immune responses as Ca^2+^ channel.

The biochemical function of plant TNLs has been revealed with their TIR domains possessing NAD(P)^+^ cleaving enzymatic activity (Horsefield et al. 2019; Wan et al. 2019). Tetrameric TNL structures in active state confirmed that tetramerization in the TIR domain creates the active site for catalysis (Ma et al. 2020; Martin et al. 2020). Effector-activated TNLs function as weak NAD(P)^+^ cleaving enzyme to produce small signaling molecule instead of causing NAD(P)^+^ depletion to induce cell death (Wan et al. 2019). Upon activation, TNL signaling converges on the lipase-like proteins Enhanced Disease Susceptibility1 (EDS1), Senescence-Associated Gene101 (SAG101) and Phytoalexin Deficient4 (PAD4) (Wagner et al. 2013). In Arabidopsis, AtEDS1 heterodimerizes with AtPAD4 and also functions with helper NLR AtADR1s to mediate bacterial growth restriction and resistance, while AtEDS1 heterodimerizes with AtSAG101 and also functions with helper NLR AtNRG1s to control cell death (Lapin et al. 2019). Over-expression of ADR1 and NRG1 can cause cell death in *N. tabacum* independently, suggesting that they could be the ultimate arbiter of immunity in TNL signaling pathway (Collier et al. 2011).

In a parallel landmark study, Jacob et al. demonstrated that active AtADR1 and AtNRG1 also function as calcium-permeable cation channel for cell death function (Jacob et al. 2021). First, the authors resolved the 4HB structure of AtNRG1.1 CC^R^ domain and found it similar to both ZAR1 CC and the cell-death domain of animal MIXED-LINEAGE KINASE-LIKE (MLKL). Animal MLKL has been shown to function as cation channel (Xia et al. 2016). Activation mechanisms of AtNRG1 and AtADR1 by TNL enzymatic activity directly or via EDS1/SAG101/PAD4 remain elusive. Hence Jacob et al. used auto-active AtNRG1.1 allele (D485V) and wide-type AtADR1 that can cause autonomous cell death in *Nb* to study their channel activity. AtNRG1.1 D485V and AtADR1 oligomerize, enrich in PM, and induce Ca^2+^ influx to cause cell death in *Nb* and human HeLa cells, and the cell death activity can be suppressed by Ca^2+^ channel blockers such as LaCl3 and GdCl3 (Jacob et al. 2021). Similar to ZAR1, mutation of negative charged residues in the very N-terminal region of AtNRG1.1 D485V (E14Q) and AtADR1 (D11N) led to reduced Ca^2+^ influx and delayed cell death. Electrophysiology study in human HEK293 cells confirmed that AtNRG1.1 D485V functions as non-selective calcium-permeable cation channel (Jacob et al. 2021). In Arabidopsis, all TNL immune receptors tested so far require the redundant RNLs of the ADR1 and NRG1 subfamilies (Jubic et al. 2019). Hence the authors propose that TNL activation induces RNL-dependent Ca^2+^ influx, to initiate cell death and, likely, immune responses.

The two landmark studies combined reveal the molecular and biochemical mechanisms of how the two major classes of plant intracellular immune receptors, CNLs and TNLs, control cell death and immune response by activating relevant NLR (ZAR1 of CNL by itself and RNLs downstream of TNLs) as Ca^2+^ channel. ZAR1 represents a special class of CNLs functioning as both sensor of the pathogen and executor of immune responses as Ca^2+^ channel (Wang et al. 2019a; Bi et al. 2021). In Arabidopsis, unlike ZAR1, some CNLs such as RPS2 and RPS5, require ADR1s as helper NLR for full function (Saile et al. 2020). To confer resistance, RPS2 and RPS5 also require PM-localized integrin-like protein NDR1 (NON-RACE-SPECIFIC DISEASE RESISTANCE1) whose function remains elusive (Saur et al. 2021). Hence it requires future efforts to define if other CNLs also function as Ca^2+^ channel or alternative signaling mechanisms exist for CNLs. In *Nb*, the helper class NRCs are required for phylogenetically related CNLs to activate cell death and immune responses (Wu et al. 2017). Hence, it remains to investigate whether NRCs function as Ca^2+^ channel but not those CNLs that require NRCs to transduce signal. Jacob et al. used auto-active RNL alleles of AtNRG1.1 D485V and AtADR1 to demonstrate their channel activities. In the biological relevant context of ETI, whether effector activated TNL signaling pathway involving EDS1/SAG101/PAD4 also leads to RNL functioning as Ca^2+^ channel remains to be demonstrated.

Another important open question is how CNL- and RNL-mediated Ca^2+^ influx controls cell death and immunity. Cell death is either a consequence of Ca^2+^ cytotoxicity or a product of Ca^2+^-responsive factors that execute a cell death program. Cell death and defense activation can be uncoupled during ETI (Lapin et al. 2019; Laflamme et al. 2020). In the TNL signaling pathway, the EDS1/SAG101/NRG1 branch mainly controls cell death, while the EDS1/PAD4/ADR1 branch dominates defense activation (Lapin et al. 2019). How do NRG1- and ADR1-mediated Ca^2+^ influxes initiate different outputs? What are their Ca^2+^-responsive factors respectively? The requirement of ZAR1 residue E11 specially involved in its Ca^2+^ channel activity for cell death, ROS accumulation and bacterial growth restriction suggests that ZAR1-mediated Ca^2+^ influx controls both cell death and defense activation (Wang et al. 2019a; Bi et al. 2021). How exactly are CNL ZAR1-mediated cell death and defense inter-connected and separated? Do CNLs and RNLs share Ca^2+^-responsive factors for signaling? Answering these questions raised from the two recent landmark studies will substantially advance our understanding of plant NLR functions.

## Data Availability

Not applicable.
